# Efficacy of Sleeve Gastrectomy with Concomitant Hiatal Hernia Repair versus Sleeve–Fundoplication on Gastroesophageal Reflux Disease Resolution: Systematic Review and Meta-Analysis

**DOI:** 10.3390/jcm12093323

**Published:** 2023-05-06

**Authors:** Lidia Castagneto-Gissey, Maria Francesca Russo, Vito D’Andrea, Alfredo Genco, Giovanni Casella

**Affiliations:** Department of Surgery, Sapienza University of Rome, Viale Regina Elena, 324, 00161 Rome, Italy; lidia.castagnetogissey@uniroma1.it (L.C.-G.);

**Keywords:** GERD, hiatal hernia repair, sleeve gastrectomy, bariatric surgery, Nissen sleeve, fundoplication

## Abstract

(1) Background: There is still disagreement over how sleeve gastrectomy (SG) affects gastroesophageal reflux disease (GERD). The debate regarding the best option for patients undergoing bariatric surgery who are also affected by GERD and/or hiatal hernia continues to divide the community of bariatric surgeons. While concomitant hiatal hernia repair (SG + HHR) has been proposed as a means of reducing the risk of GERD following SG with varying degrees of success, the addition of a fundoplication (SG + FP) has been suggested in recent years as a way to improve the lower esophageal sphincter’s competency. The aim of this study is to systematically review and meta-analyze the efficacy of SG + HHR versus SG + FP on GERD remission in patients with obesity. (2) Methods: A systematic review of the literature was conducted, and studies analyzing the effects of SG + HHR versus SG + FP on postoperative GERD were included. The methodological quality of included trials was evaluated. The primary outcome was postoperative GERD rate, erosive esophagitis, and 12-month weight loss. Secondary outcomes included postoperative complications and mortality. The PRISMA guidelines were used to carry out the present systematic review (PROSPERO Registration Number: CRD42023405600). (3) Results: Fifteen articles with a total of 1164 patients were included in the meta-analysis; 554 patients underwent SG + HHR while 610 underwent SG + FP. In the SG + HHR group, 58.5 ± 28.9% of subjects presented clinical GERD symptoms compared to 20.4 ± 17.5% postoperatively (*p* < 0.001). In the SG + FP group, 64.8 ± 39.4% were affected by GERD preoperatively compared to only 5 ± 8.1% postoperatively (*p* < 0.001). SG + FP patients had a significantly greater GERD remission compared to SG + HHR (*p* < 0.001). Weight loss was similar between groups (*p* = 0.125). The rate of leaks was 0.18% and 0.33% in the SG + HHR and SG + FP, respectively (*p* = 0.657), while perforations were significantly higher after SG + FP compared to the SG + HHR group (3.1% versus 0%, *p* = 0.002). The mortality rate was significantly greater in the SG + FP group (0.5% versus 0%, *p* = 0.002). (4) Conclusions: This study revealed that both SG with concomitant HHR and sleeve–fundoplication are effective in terms of reflux resolution and weight outcomes, with superiority of SG + FP in terms of GERD control, despite a greater overall complication rate. Both strategies can therefore be suggested as a suitable alternative variant to a conventional SG in subjects with obesity and concomitant hiatal hernia and/or GERD. Studies with extended follow-up and direct comparisons of these surgical approaches to conventional SG are warranted.

## 1. Introduction

Sleeve gastrectomy (SG) continues to be the most popular bariatric procedure around the world, accounting for an estimated 67% of all primary bariatric operations performed globally [1].

Although SG has been widely proven to be considerably effective in terms of weight loss and comorbidity resolution rates [2,3,4,5], there is still disagreement over how SG affects gastroesophageal reflux disease (GERD). The remission of GERD symptoms has not been definitively linked to the post-bariatric resolution of obesity. In fact, the sort of bariatric procedure chosen is what is at play in this mechanism. Indeed, it has been demonstrated that Roux-en-Y gastric bypass (RYGB) is most effective at improving or eliminating GERD [6]. On the contrary, some authors have found a decrease in de novo GERD after SG [7,8], while others have shown an exacerbation or increased incidence of de novo GERD symptoms following this surgical procedure [9,10,11].

In both the general population and after SG, hiatus hernia is thought to be a major risk factor for GERD. About 40% of subjects affected by morbid obesity have a variable degree of hiatal hernia [12], and its frequency rises following SG [9,13]. The debate regarding the best option for obese patients undergoing bariatric surgery who are also affected by GERD and/or hiatal hernia continues to divide the community of bariatric surgeons. The majority of high-volume sleeve surgeons advise actively seeking for and fixing hiatal hernias [7,8] when carrying out SG. Other surgeons agree that obese patients with GERD and/or hiatus hernia should not be given the option of SG and should instead have an RYGB. If these patients do receive SG, there is no agreement on whether they should also have their hiatal hernia repaired at the same time. In order to limit the likelihood of postoperative reflux, many surgeons suggest that SG is only safe, in obese patients with hiatal hernia, when used in conjunction with concomitant closure of hiatal defects [14,15]. However, there is not yet a consensus [14] on whether this is actually advantageous or on the method of closure of the hiatal defect.

While crural repair has been proposed as a means of reducing the risk of GERD following SG with varying degrees of success, the addition of a fundoplication has been suggested in recent years as a way to improve the competency of the lower esophageal sphincter (LES). The so-called “sleeve–fundoplication” (SG + FP) has been described through various surgical techniques, although Rossetti, Collis–Nissen, and Nissen fundoplication are the most commonly performed variants. Nevertheless, published research in this regard is extremely limited, and the evidence is quite conflicting.

There is no systematic review currently available evaluating the efficacy and technical aspects of concomitant SG with hiatal hernia repair (SG + HHR) versus SG + FP for the treatment of GERD in patients affected by severe obesity and eligible for SG. Therefore, the aim of this study is to systematically review and meta-analyze the efficacy of concomitant SG + HHR or SG + FP on GERD remission and postoperative outcomes in patients with obesity.

## 2. Materials and Methods

### 2.1. Search Strategy and Selection of Trials

This study provides a systematic review and meta-analysis of previously published data, which was carried out according to the Preferred Reporting Items for Systematic Reviews and Meta-Analyses (PRISMA Statement) criteria [16]. This study was registered to the PROSPERO International prospective register of systematic reviews (Registration Number: CRD42023405600). The PICO strategy was used to formulate the guiding question: “What are the effects of sleeve gastrectomy with concomitant hiatal hernia repair or fundoplication on postoperative GERD?” [17]. The search was performed using the following electronic databases without any year restriction, from inception through 28 February 2023: PubMed, Embase, the Web of Science Core Collection, and the Cochrane Central Register of Controlled Trials. All abstracts in the English language were screened for applicability. A manual search using the following keywords extracted from the Medical Subjects Heading (MeSH) was performed: (“bariatric surgery” OR “obesity surgery” OR “weight loss surgery” OR “metabolic surgery” OR “Sleeve gastrectomy” OR SG OR LSG) AND (“Hiatus Hernia” OR “Hiatal Hernia” OR “Esophageal Hernia” OR “Paraesophageal Hiatal Hernia”) AND (“Gastroesophageal Reflux” OR “Gastro-esophageal Reflux Disease” OR “Gastro Esophageal Reflux Disease” OR “Gastro-oesophageal Reflux” OR “Gastro-oesophageal Reflux” OR “Gastroesophageal Reflux Disease” OR “Esophageal Reflux” OR GERD) AND (obesity OR overweight OR obese).

The eligibility criteria for the selection of articles, according to the PICO strategy, were as follows: prospective or retrospective cohort studies with adults aged 18 years or over (population); performance of sleeve gastrectomy with concomitant hiatal hernia repair (intervention); comparison with sleeve–fundoplication (comparison); incidence of GERD, degree of weight loss, postoperative complications (outcomes). The studies excluded were those not written in English and those that did not provide the full online abstract.

All articles analyzing pre- and postoperative GERD and/or hiatal hernia incidence, regardless of the presence or absence of preoperative reflux symptoms, were included. The focus was on postoperative GERD rather than the preoperative existence of this symptom. Indeed, GERD can be often asymptomatic despite the fact that endoscopic esophageal erosions can be present anyways, or, on the contrary, reflux can also be non-erosive (NERD). Furthermore, SG itself can cause the development of de novo GERD or a worsening of pre-existing GERD.

Two independent reviewers (LCG, MFR) screened and selected the studies to be included in the review. Conflicts were handled by consensus, and an adjudicator (GC) was consulted when necessary. Only studies that were fully available and designed to evaluate the effects on GERD of hiatal hernia repair or fundoplication during sleeve gastrectomy and assessing weight loss and postoperative complications were included.

### 2.2. Outcome Measures

The primary outcome was the rate of postoperative GERD symptoms, erosive esophagitis, and 12-month weight loss. Secondary outcomes included postoperative complications and mortality.

### 2.3. Inclusion and Exclusion Criteria

Articles were considered eligible for inclusion if they met the following criteria: (1) the articles reported outcomes for sleeve gastrectomy and GERD/hiatus hernia, (2) the publication described preoperative and postoperative GERD symptoms, and (3) available data could be extracted from studies to calculate outcomes.

If similar studies adopted data from overlapping populations, only the study with the most comprehensive information was included. Studies evaluating GERD outcomes after concomitant SG with HHR or after sleeve–fundoplication (regardless of the technical variant) with or without a comparison group of patients undergoing SG alone and including follow-up duration, weight loss outcomes, and postoperative complications and mortality were included.

Animal studies, case reports, conference abstracts, comments, reviews, guidelines, studies with less than 10 patients, and studies with less than one year of follow-up were excluded.

### 2.4. Critical Assessment of Trials and Collection of Data

Two independent reviewers evaluated the methodological quality of eligible studies using validated scales; in the event of a disagreement, the final score was decided by consensus.

The Newcastle–Ottawa scale (NOS) was used to rate quality assessment for non-randomized trials [18]. The NOS consists of three parts: selection (0–4 points), comparability (0–2 points), and outcome assessment (0–3 points). Scores of 7–9 points were assigned as high-quality studies. The quality of randomized controlled trials was evaluated with the Jadad scale tool [19]. It consists of three domains: methods to generate randomization sequences (0–2 points), double-blinding (0–2 points), and withdrawal and dropouts (0–1 points). Studies with a Jadad score of 4 or more were defined as high quality. Two authors (MFR and LCG) separately assessed the included studies, and discrepant opinions between authors were obtained by discussion and consensus.

### 2.5. Data Extraction

The two reviewers (MFR and LCG) independently gathered data, which they then compared, and they cross-checked articles based on the inclusion and exclusion criteria. Missing data were sought in the journal’s database and included if present. All studies with missing text or with insufficiently reported data were excluded.

The following data were independently retrieved using a pre-selected data extraction form for each study: publication year, country, sample size, type/modality of study, dropouts, demographics, type of surgical procedure, outcomes of interest, follow-up duration.

### 2.6. Statistical Analysis

All statistical analyses were performed by MedCalc (v20.211) [20] and Meta-Mar (v3.5.1). Meta-analyses were performed using odds ratio (OR) for the dichotomous outcome, while the mean difference (MD) or standardized mean difference (SMD) was used for continuous outcome measures, depending on whether or not the same scales measured the outcomes. The heterogeneity among the studies was checked using Cochrane’s Q [21] and the I2 statistical tests [22,23]. The model of random effects was adopted for the analysis.

Furthermore, we also used Begg’s and Egger’s tests for assessing possible publication bias (*p* < 0.10 was considered significant) [24,25].

Analysis of variance (ANOVA) was used to test differences in %EWL in patients assigned either to SG + HHR or SG + FP [26]. Furthermore, 95% CI was calculated between the two groups.

## 3. Results

**Figure 1:**
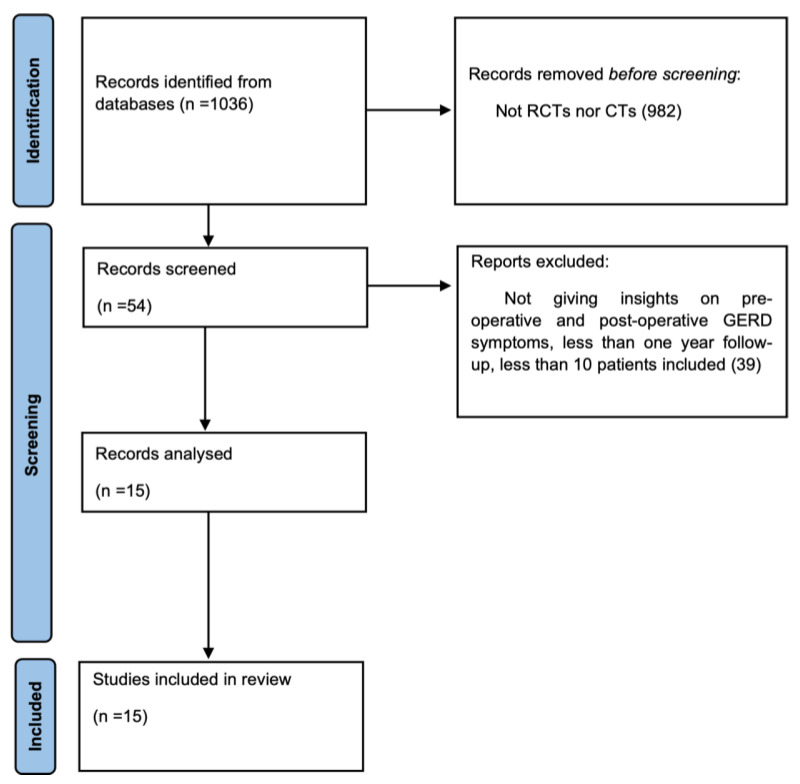
Flowchart of article selection according to the PRISMA guidelines.

A total of 1036 studies were found in the electronic search. After reviewing titles and abstracts, 982 were not randomized clinical trials or clinical trials and were excluded. The remaining 54 articles were analyzed, and 39 were excluded because did not give insights on preoperative and postoperative GERD symptoms, had less than one year of follow-up, or had a cohort presenting less than 10 patients. Thus, 15 articles were included in the final analysis (Figure 1).

Nine studies (60%) were retrospective, five (33.3%) were prospective, and one (6.7%) was an RCT. A total of 1164 patients participated in the selected studies. Five hundred fifty-four patients underwent SG + HHR while 610 underwent SG + FP (Table 1 and Table 2). Mean follow-up was 37.3 ± 28.1 months after SG + HHR and 17.4 ± 9.3 months after SG + FP.

The majority of studies (11 out of 15) reported a very high prevalence of GERD, hiatal hernia, or both preoperatively with only some exceptions [10,16,17,23].

In the SG + HHR group, all patients were affected by symptomatic GERD or hiatal hernia either diagnosed preoperatively by endoscopy, manometry, and upper g-i contrast study or intraoperatively for which the authors deemed it necessary to add a cruroplasty whilst performing SG. With regard to the SG + FP group, six studies added a fundoplication to SG due to a high prevalence of symptomatic GERD, while only one study performed SG + FP as part of their study design (i.e., randomized comparative analysis between SG alone and SG + FP) [23].

The definition of GERD was quite variable. This was supplied by the majority of authors. Most articles defined GERD, based on reported symptoms, as the presence of heartburn or regurgitation, evaluating its severity by using scales or scores and the necessity of using antacids or proton pump inhibitors. Only five studies [13,15,16,18,23] actually used manometry, endoscopy, or upper g-i contrast study to diagnose GERD.

### 3.1. Methodological Quality Assessment and Risk of Bias

Methodological quality for non-randomized trials was evaluated using the NOS scale. In the SG + HHR group, three (33.3%) were prospective and six (66.6%) were retrospective cohort studies with a high quality assessment according to NOS in all cases (two studies scored 9, five scored 8, and three scored 7 points) (Table 1). Methodological quality evaluated using Jadad’s validated scale revealed just one (14.3%) randomized controlled trial in the SG + FP group with a score of 5, indicating a high quality of the study design in terms of randomization sequence, blinding, and dropouts. With regard to the other included studies in the SG + FP group, four (57.1%) were retrospective and two (28.6%) were prospective studies with an overall high methodological quality (one rated 9, three rated 8, two rated 7 points) (Table 2).

The *p* values for Egger’s and Begg’s tests for GERD in patients undergoing SG + FP were 0.0023 and 0.3476, respectively. For what concerns BMI, *p* values were *p* = 0.8657 and *p* = 0.00. Furthermore, the *p* values for Egger’s and Begg’s tests for GERD in patients assigned to SG + HHR were *p* = 0.0012 and *p* = 0.0.008, while for BMI in the same group, these values were *p* = 0.2320 and *p* = 0.1765 (Supplementary Figures S1 and S2).

### 3.2. Primary Outcomes

**Figure 2:**
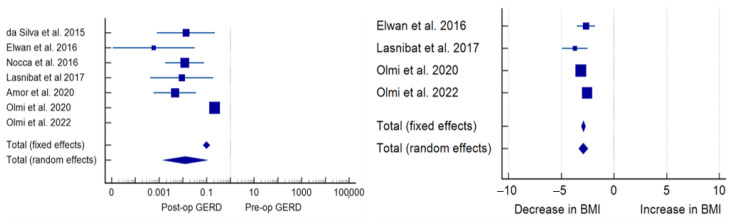
Forest plot of SG + FP effects on GERD (**left panel**) and BMI (**right panel**). Plot of the measure of effect for each of the studies (square) incorporating confidence intervals represented by horizontal lines. The area of each square is proportional to the study’s weight in the meta-analysis [11,18,19,20,21,22,23].

**Figure 3:**
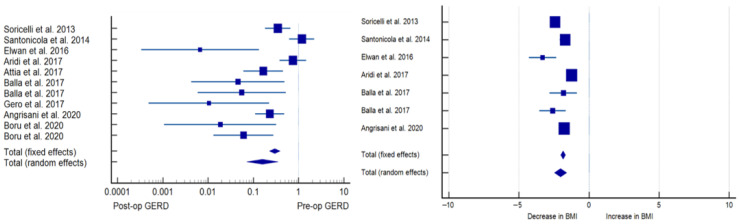
Forest plot of SG + HHR effects on GERD (**left panel**) and BMI (**right panel**) [9,10,11,12,13,14,15,16,17].

Patients assigned to both SG + HHR and SG + FP had substantial GERD remission compared to preoperative levels. In the SG + HHR group, 58.5 ± 28.9% of subjects presented clinical GERD symptoms compared to 20.4 ± 17.5% postoperatively (*p* < 0.001). In the SG + FP group, 64.8 ± 39.4% were affected by GERD preoperatively compared to only 5 ± 8.1% postoperatively. SG + FP patients had a significantly greater GERD remission compared to SG + HHR (*p* < 0.001) (Table 3 and Table 4, Figure 2 and Figure 3).

Analysis of variance between the two groups, namely SG + HHR and SG + FP, showed a non-statistically significant difference in terms of %EWL (*p* = 0.125, 95% CI −17.78 to 8.31). There was a mean postoperative BMI of 31.9 and 30.7 kg/m^2^ (*p* = 0.564) and a %EWL of 63.7 and 68.7% (*p* = 0.125) after SG + HHR and SG + FP, respectively.

### 3.3. Secondary Outcomes

Major postoperative complications included intra- or postoperative bleeding, gastric perforation, staple-line leak, and mortality (Table 5). The rate of overall complications mainly related to gastric wrap perforation and consequent reoperations was greater after SG + FP compared to SG + HHR (*p* = 0.002). Gastric valve perforation was the most frequently reported indication for reoperation after SG + FP with an overall rate of 3.1%. The rate of leaks was 0.18% and 0.33% in SG + HHR and SG + FP, respectively (*p* = 0.657), while perforations were significantly higher after SG + FP compared to SG + HHR group (3.1% versus 0%, *p* = 0.002)

The mortality rate was significantly greater in the SG + FP group (0.5% versus 0%, *p* = 0.002).

### 3.4. Subgroup Analysis

Only a limited number of articles performed a study including a control group for comparison. Specifically, two studies (10, 12) compared SG + HHR versus SG alone, two studies (20, 23) compared SG + FP versus SG alone, and two studies compared SG + simple HHR versus SG + mesh HHR (14, 17). Only 1 study (11) directly compared SG + HHR versus SG + FP.

Elwan et al. (11) compared SG + HHR (Group A) and SG + FP (Group B). Despite a small sample size (20 patients per group), the authors report a significantly greater persistence of GERD postoperatively in Group A compared to Group B (20% versus 0%, *p* = 0.035) with a recurrence of hiatal hernia in 40% of patients in Group A versus 0% in Group B.

**Figure 4:**
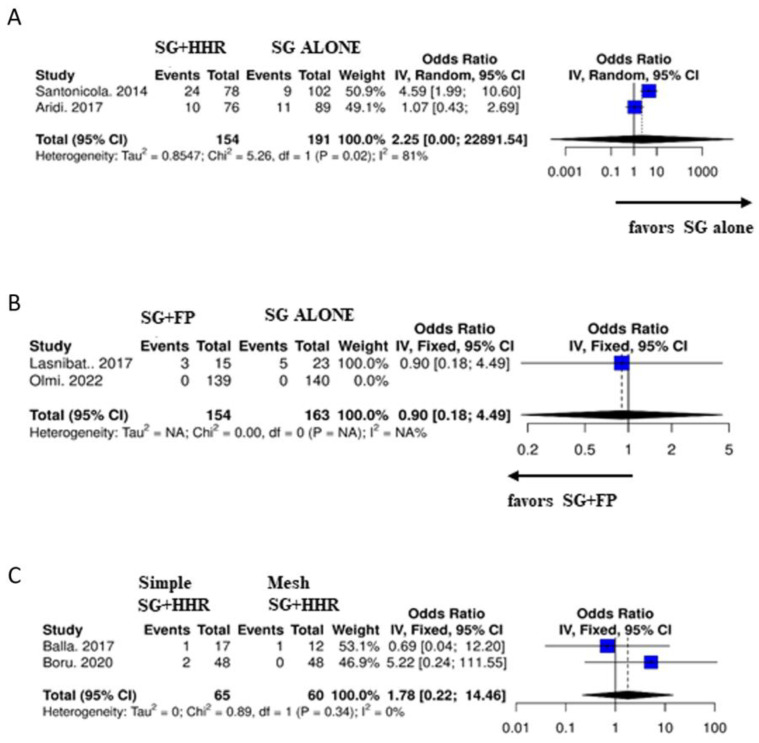
Subgroup analyses for studies with control groups. Forest plots for (**A**) SG + HHR versus SG alone [10,12]; (**B**) SG + FP versus SG alone [20,23] and (**C**) SG + simple HHR versus SG + mesh HHR [14,17]. Plot of the measure of effect for each of the studies (square) incorporating confidence intervals represented by horizontal lines. The area of each square is proportional to the study’s weight in the meta-analysis.

Subgroup analyses were carried out in those studies in which a control group was present (Figure 4). In terms of GERD remission, in the SG + HHR versus SG alone group comparison, the analysis favors SG alone (*p* = 0.02); in the SG + FP versus SG alone group comparison the analysis favors SG + FP. Furthermore, no statistically significant differences were found between the two groups when comparing simple SH + HHR versus mesh SH + HHR (*p* = 0.34).

## 4. Discussion

The need for adding anti-reflux mechanisms when performing SG in patients affected by obesity with concomitant GERD and/or hiatal hernia is still a controversial subject in current literature. GERD incidence after SG has been reported by numerous authors at extremely variable rates. Particularly in patients with a history of clinical or latent GERD, it is important to carefully choose the best bariatric procedure. The worsening or de novo onset of postoperative GERD may be caused by a number of reasons, even if a careful preoperative patient selection is completed. The following have been suggested as potential influencing factors: decreased LES pressure, delayed gastric emptying, partial division of the Helvetius fibers, blunting of the His angle, reduced gastric compliance/volume, and raised gastric pressure [9,11].

The present study comprehensively reviewed and identified all articles assessing the impact of SG + HHR or SG + FP on reflux, weight outcomes, and postoperative complications, quantitatively analyzing the presently available evidence in order to further clarify this debate.

Regarding the optimal bariatric procedure for patients with symptomatic GERD and/or hiatal hernia, most surgeons are still in strong disagreement. Although a large portion of surgeons believe that RYGB is the best choice for obese patients with GERD and/or hiatal hernia, for a variety of surgeon- or patient-related reasons, SG is often preferred. For this purpose, an attempt at identifying an alternative variant to a conventional SG is necessary.

This review provides evidence regarding the efficacy of both SG + HHR and SG + FP, with substantially improved results in terms of postoperative GERD rates and overall weight loss compared to preoperative levels (*p* < 0.001). Comparative analysis showed a greater rate of GERD remission in the SG + FP group (*p* < 0.001). The superiority of SG + FP in terms of GERD symptom remission might be attributable to the greater pressure of the gastric wrap exerted at the level of the LES, creating an anti-reflux valve, together with the possibility of avoiding or considerably reducing the risk of intrathoracic migration of the sleeved stomach. Despite the preservation of the gastric fundus, weight loss was similar between groups both in terms of postoperative BMI and %EWL.

More specifically, SG + HHR leads to a substantial decrease in GERD symptoms with respect to baseline (*p* < 0.001). An objective outcome was the incidence of erosive esophagitis revealed by pre- and postoperative endoscopy, which was reported only in two studies [27,28]. Those studies evaluating esophagitis by endoscopy found a considerable reduction in esophageal inflammatory lesions after SG + HHR. Although not all reports included this evaluation and overall information was scarce, homogeneity among studies was high and the pooled analysis showed convincing results regarding the beneficial effect of HHR coupled with SG. Only one [14] out of all the studies considered in this review reported poor outcomes following SG + HHR and avoided recommending it. Quality of life as evaluated by GERD-HRQL questionnaires was included only in two studies [28,29] showing a significant improvement compared to preoperative values with high satisfaction levels after SG + HHR. Those studies comparing SG alone with SG and concomitant HHR found differing rates of GERD remission between groups. Soricelli et al. found substantial improvement or remission of GERD after SG + HHR compared to SG alone (80.4% versus 57.9%) with de novo GERD appearing in 22.9% after SG alone and in none of the patients with HHR [30]. Aridi et al. found no substantial difference between the two groups [31], while Santonicola et al. surprisingly highlighted a significant decrease in the prevalence of typical GERD symptoms only in the conventional SG group [14]. The study by Elwan et al. was the only one directly and actively comparing SG + HHR (Group A) and SG + FP (Group B). Despite a small sample size (20 patients per group), authors found a significantly greater persistence of GERD postoperatively in Group A compared to Group B (20% versus 0%, *p* = 0.035) with a recurrence of hiatal hernia in 40% of patients in Group A versus 0% in Group B. Although reflux outcomes were superior after SG + FP, Elwan et al. report a significantly greater weight loss after SG + HHR (37.9 versus 35.0 kg/m^2^, *p* = 0.001) [32].

Literature data regarding SG + FP are visibly lacking, especially when compared to SG + HHR, and evidence only derives from retrospective observational studies except for one randomized controlled trial. Nevertheless, available data seem to indicate that SG + FP is also capable of generating a significant improvement in reflux, erosive esophagitis, and weight-related outcomes compared to baseline (*p* < 0.001), despite a remarkable rate of overall complications mainly related to gastric wrap perforation and consequent reoperations compared to SG + HHR (*p* = 0.002). Worryingly, the mortality rate was also significantly greater in the SG + FP group (0.5% versus 0%, *p* = 0.002).

Gastric valve perforation was the most frequently reported indication for reoperation after SG + FP with an overall rate of 3.1%. Laparoscopic revision typically involved resecting the gastric valve, draining perigastric abscesses if present, and switching to a regular SG.

Gastric perforation after SG + FP is a completely distinct event from a gastric leak. In fact, the rate of leaks was 0.18% and 0.33% in SG + HHR and SG + FP, respectively (*p* = 0.657), while perforations were significantly higher after SG + FP compared to the SG + HHR group (3.1% versus 0%, *p* = 0.002), indicating how such complications are the result of different pathophysiological events. Numerous theories have been put forth, including incongruous manipulation of the gastric fundus during fundoplication, thermal injury, and insufficient vascularization of the gastric valve [33,34]. This result must be interpreted with caution since it may be impacted by different surgical approaches, level of surgeon expertise, valve architecture, outcome reporting, patient comorbidities, postoperative complication definition, and patient selection bias.

Due to the potential influence of a learning curve phase in a novel, non-standardized, and experimental technique such as SG + FP, these results should be interpreted with caution.

Only two studies compared SG alone with SG + FP [32,35] and found GERD recurrence or persistence in a significantly greater proportion of subjects in the former group (20% versus 0%, respectively, *p* = 0.035) [29], with a substantial reduction in erosive esophagitis in the latter group (23.4% versus 2%, respectively, *p* = 0.002) [35].

With regard to weight outcomes, the change in BMI after SG + FP was similar to SG + HHR, and both were superimposable to reported weight loss for conventional SG [36]. Leaving a portion of the gastric fundus may undermine the weight-loss impact in patients undergoing SG + FP compared to those receiving SG + HHR, raising concerns about potential weight gain in the longer term. Cautious interpretation of these results is likewise necessary due to potential confounders linked to the use of variable bougie sizes, dietary regimen compliance, and limited long-term follow-up that prevent the creation of robust and conclusive findings.

According to scientific literature, the precise prevalence of postoperative GERD in patients with obesity and hiatal hernia, if SG was performed without HHR or FP, is presently unknown, and studies that directly compare these two methods are missing. Nevertheless, a very limited number of bariatric surgeons would recommend performing SG in a patient with a hiatal hernia without repairing the defect. Furthermore, intrathoracic migration of the gastric sleeve, which occurs at a rate of approximately 7% following SG [37], is a significant complication that could result in both recurring and new onset of GERD. The primary reason for intrathoracic migration may be the failure to repair hiatal hernias intraoperatively, emphasizing how crucial it is to precisely locate and treat hiatal hernias during SG. This was also confirmed by the latest consensus statement on SG, where most surgeons advise aggressive exploration of the crural area in order to identify and repair hiatal hernias if present [38].

The majority of authors support posterior crural approximation as a closure method. Due to the restoration of the normal anti-reflux gastroesophageal angle, the posterior repair is recognized as being better for the anti-reflux mechanism. It should come as no surprise that despite the use of a range of suture materials including silk, Ethibond, and Prolene, every single author advocated the use of non-absorbable sutures. Large hiatal defects were also reinforced using a biological mesh.

Likewise, the selection of the fundoplication type should be taken into account as a potential cause of selection bias and heterogeneity because it may affect results. Future research should concentrate on this comparison because there are insufficient data to endorse one fundoplication over another.

However, when comparing GERD remission between SG and RYGB, a large cohort study comparing the two procedures including a total of 38,699 patients found only 15.9% of patients with GERD who underwent SG experienced remission, compared to 62.8% of patients who received RYGB [10]. Correspondingly, reflux remission was observed to be 25% and 60.4%, respectively, in a 5-year randomized controlled trial comparing SG and RYGB [39]. Considering such an evident superiority of RYGB in terms of the prevalence of reflux remission in subjects with obesity and GERD, it appears reasonable to select this treatment option in this subpopulation when possible.

Nevertheless, heartburn may be reported by certain individuals with esophageal hypersensitivity or functional abnormalities that are not supported by a true pathologic reflux. In fact, the correlation between symptoms and esophagitis is not a sensitive marker for pathologic GERD [40,41]. This could explain why not all patients, but only two in three subjects receiving RYGB, experience a remission of GERD symptoms. Since these results are susceptible to criticism, it would be preferable to collect more reliable evidence in the future by objective data assessment using pH–impedance 24 h monitoring in conjunction with esophageal manometry and upper endoscopy [11,41].

Although RYGB is widely considered to be the best and most widely implemented surgical option for the treatment of GERD following SG, other possible surgical conversions have been successfully proposed to resolve this common post-SG condition. Single anastomosis sleeve ileal bypass (SASI) and Santoro transit bipartition, where a gastro-ileal anastomosis at the level of the antrum in either a loop or a Roux-en-Y configuration, is performed, respectively, have been described to improve GERD in an elevated proportion of patients. In fact, a recent meta-analysis found a 92% remission of GERD after SASI [42], while reported evidence is still quite limited regarding Santoro’s procedure.

Although SG + HHR and SG + FP are both successful in the short term for weight reduction, GERD remission, esophagitis resolution, and discontinuation of PPI therapy, more research is necessary to examine their impact in the medium and long term through the use of objective instrumental examinations. SG + FP needs to be approached with caution due to its more recent nature and limited evidence, especially in the mid–long term, while well-designed randomized trials comparing both procedures in the future are necessary.

### Study Strengths and Limitations

This study’s strength is that it is currently the first systematic review and meta-analysis evaluating the differences in reflux and weight outcomes in patients undergoing either SG + HHR or SG + FP, providing insights and shedding light on the possible benefits and weaknesses of each procedure.

Some limitations must be acknowledged in the present study. The majority of included articles focused their conclusions on symptoms rather than objective assessment, which could result in an incorrect and overestimated GERD diagnosis. Another important factor contributing to clinical variability is the diversity of surgical techniques for both hiatal hernia repair (anterior, posterior, or mesh repair) and fundoplication (Nissen, Nissen-Collis, or Rossetti). Further shortcomings of this study are that most of the articles reviewed were retrospective in nature, and possible inconsistencies could have arisen from differences in diagnosis and classification of hiatal hernia or GERD. Finally, the results were probably impaired by additional bias (mostly small-trial bias), and only a few studies were sufficiently powered to address this problem.

Given the aforementioned drawbacks, additional high-quality studies with longer follow-ups should be carried out in the future to demonstrate the impact of SG + HHR or SG + FP on GERD in order to reach more conclusive evidence.

## 5. Conclusions

This study revealed that both SG with concomitant HHR and sleeve–fundoplication are effective in terms of reflux resolution and weight outcomes, with superiority of SG + FP in terms of GERD control, despite a greater overall complication rate. Both strategies can therefore be suggested as a suitable alternative variant to a conventional SG in subjects with obesity and concomitant hiatal hernia and/or GERD.

Studies with extended follow-up and direct comparisons of these surgical approaches to conventional SG are warranted.

## Figures and Tables

**Table 1:** Characteristics of studies assessing SG + HHR.

Authors	Year of Publication	Study Type	Control Group	Number of Patients	Follow-Up (Months)	Surgical Technique	Quality Assessment
Soricelli et al. [9]	2013	Prospective	No	97	18	Posterior repair using non-absorbable sutures	9
Santonicola et al. [10]	2014	Retrospective	Group A (SG + HHR) vs. Group B (SG alone)	78 vs. 102	14.6	Posterior repair using 0-Ethibond	9
Elwan et al. [11]	2016	Retrospective	Group A (SG + HHR) vs. Group B (SG + FP)	20 vs. 20	14.1	Posterior repair using 2–0 non-absorbable sutures	8
Aridi et al. [12]	2017	Retrospective	Group A (SG + HHR) vs. Group B (SG alone)	76 vs. 89	12	Posterior repair using 2–0 Ethibond sutures	8
Attia et al. [13]	2017	Prospective	No	53	18	Posterior repair using 0-Ethibond	8
Balla et al. [14]	2017	Retrospective	Group A (SG + simple HHR) vs. Group B (SG + mesh HHR)	12 vs. 17	33.2 ± 16.3	Posterior repair using 2–0 non-absorbable sutures vs. cruroplasty using absorbable synthetic mesh	7
Gero et al. [15]	2017	Retrospective	No	14	12.5	Posterior closure with EGJ fixed to the median arcuate ligament using 0-non-absorbable sutures	8
Angrisani et al. [16]	2020	Retrospective	No	91	94 ± 10	Posterior repair using 2–0 non-absorbable sutures	8
Boru et al. [17]	2020	Prospective	Group A (SG + simple HHR) vs. Group B (SG + mesh HHR)	48 vs. 48	59.1 ± 9.1	Posterior repair using non-absorbable sutures vs. cruroplasty using biologic mesh	7

**Table 2:** Characteristics of studies assessing SG + FP.

Authors	Year of Publication	Study Type	Control Group	Number of Patients	Follow-Up (Months)	Surgical Technique	Quality Assessment
da Silva et al. [18]	2015	Retrospective	No	122	36	Sleeve Collis–Nissen Hiatoplasty	7
Elwan et al. [11]	2016	Retrospective	Group A (SG + HHR) vs. Group B (SG + FP)	20 vs. 20	14.1	Nissen sleeve	8
Nocca et al. [19]	2016	Prospective	No	25	12	Nissen sleeve	8
Lasnibat et al. [20]	2017	Retrospective	Group A (SG + FP) vs. Group B (SG alone)	15 vs. 23	12	Nissen sleeve	7
Amor et al. [21]	2020	Prospective	No	70	12	Nissen sleeve	8
Olmi et al. [22]	2020	Retrospective	No	220	24	Sleeve Rossetti fundoplication	9
Olmi et al. [23]	2022	RCT	Group A (SG alone) vs. Group B (SG + FP)	140 vs. 138	12	Sleeve Rossetti fundoplication	5

**Table 3:** Clinical data of patients undergoing SG + HHR.

Authors	Year of Publication	Number of Patients	Pre-op BMI (kg/m^2^)	Post-op BMI (kg/m^2^)	%EWL	Pre-op GERD n (%)	Post-op GERD n (%)	Pre-op HH n (%)	Post-op HH n (%)	Pre-op Esophagitis n (%)	Post-op Esophagitis n (%)	Bleeding n (%)	Perforation n (%)	Leaks n (%)	Mortality n (%)
Soricelli et al. [9]	2013	97	44 ± 3.5	32.8 ± 5.5	NR	60 (61.9)	19 (19.5)	97 (100)	NR	56 (58)	NR	0 (0)	0 (0)	0 (0)	0 (0)
Santonicola et al. [10]	2014	78	44.6 ± 7	31.7 ± 8	62.8 ± 3.53	30 (38.4)	34 (43.3)	23 (28.9)	NR	NR	NR	0 (0)	0 (0)	0 (0)	0 (0)
Elwan et al. [11]	2016	20	45.05 ± 2.96	35.0 ± 2.99	57	20 (100)	4 (20)	5 (25)	8 (40)	NR	NR	0 (0)	0 (0)	1 (5)	0 (0)
Aridi et al. [12]	2017	76	42.7 ± 15.3	28 ± 6.6	87 ± 23.7	29 (38.2)	24 (31.9)	76 (100)	2 (2.6)	NR	19 (25)	5 (6.5)	0 (0)	0 (0)	0 (0)
Attia et al. [13]	2017	53	50.1	NR	61	47 (88.6)	30 (56.6)	NR	NR	NR	NR	1 (1.8)	0 (0)	0 (0)	0 (0)
Balla et al. [14]	2017	12	42.1 ± 8.3	29.7 ± 4.1	NR	8 (66.6)	1 (8.3)	12 (100)	2 (16.6)	4 (33.3)	NR	0 (0)	0 (0)	0 (0)	0 (0)
Balla et al. [14]	2017	17	43.5 ± 4.7	32.8 ± 3.2	NR	9 (52.9)	1 (5.8)	17 (100)	0 (0)	4 (23.5)	NR	0 (0)	0 (0)	0 (0)	0 (0)
Gero et al. [15]	2017	14	41	30.9	NR	14 (100)	3 (21.4)	12 (85.7)	NR	4 (28.5)	NR	0 (0)	0 (0)	0 (0)	0 (0)
Angrisani et al. [16]	2020	91	44.8 ± 6.1	34.9 ± 4.9	58.4 ± 15.6	36 (39.6)	12 (13.6)	37 (40.6)	15 (16.5)	22 (24)	15 (16.5)	NR	NR	NR	NR
Boru et al. [17]	2020	48	NR	NR	65–7 ± 17.1	17 (35.4)	0 (0)	11 (22.3)	0 (0)	6 (12.5)	2 (5.2)	0 (0)	0 (0)	0 (0)	0 (0)
Boru et al. [17]	2020	48	NR	NR	55.9 ± 15.1	20 (41.6)	2 (4.1)	14 (29.1)	0 (0)	4 (8.3)	2 (4.3)	0 (0)	0 (0)	0 (0)	0 (0)

**Table 4:** Clinical data of patients undergoing SG + FP.

Authors	Year of Publication	Number of Patients	Pre-op BMI (kg/m^2^)	Post-op BMI (kg/m^2^)	%EWL	Pre-op GERD n (%)	Post-op GERD n (%)	Pre-op HH n (%)	Post-op HH n (%)	Pre-op Esophagitis n (%)	Post-op Esophagitis n (%)	Bleeding n (%)	Perforation n (%)	Leaks n (%)	Mortality n (%)
da Silva et al. [18]	2015	122	42.5 ± 5.6	NR	64.4 ± 7.2	28 (23)	0 (0)	82 (67)	4 (3.3)	NR	NR	1 (0.8)	0 (0)	0 (0)	0 (0)
Elwan et al. [11]	2016	20	44.10 ± 2.48	37.95 ± 2.1	NR	20 (100)	0 (0)	6 (30)	0 (0)	NR	NR	6 (30)	0 (0)	0 (0)	1 (5)
Nocca et al. [19]	2016	25	42 ± 4.8	NR	58 ± 23	23 (92)	3 (12)	22 (88)	NR	10 (40)	0 (0)	1 (4)	1 (4)	0 (0)	0 (0)
Lasnibat et al. [20]	2017	15	33.9 ± 2.11	26.6 ± 1.7	82.02	15 (100)	3 (20)	NR	NR	12 (80)	3 (20)	0 (0)	0 (0)	0 (0)	0 (0)
Amor et al. [21]	2020	70	40 ± 5	NR	69 ±20	53 (76)	1 (0.7)	63 (90)	NR	44 (63)	14 (28.6)	1 (0.7)	0 (0)	1 (0.7)	0 (0)
Olmi et al. [22]	2020	220	42.58 ± 5.93	29.4	70.1	137 (62.3)	2 (0.9)	62 (28.2)	NR	65 (29.5)	2 (0.9)	6 (2.7)	12 (5.5)	1 (0.5)	1 (0.45)
Olmi et al. [23]	2022	138	43.4 ± 5.9	29.4 ± 5.0	NR	0 (0)	0 (0)	18 (13.4)	23 (16.7)	NR	3 (2.2)	1 (0.7)	6 (4.3)	0 (0)	1 (0.72)

**Table 5:** Cumulative incidence of postoperative complications per group.

	SG + HHR (n = 554)	SG + FP (n = 610)	*p* Value
Bleeding, n (%)	6 (1.08)	10 (1.63)	0.07
Gastric perforation, n (%)	0 (0)	19 (3.1)	0.002
Staple-line leak, n (%)	1 (0.18)	2 (0.33)	0.657
Mortality, n (%)	0 (0)	3 (0.5)	0.002

## Data Availability

Raw data are available upon reasonable request to the corresponding authors.

## Supplementary Materials

The following supporting information can be downloaded at: https://www.mdpi.com/article/10.3390/jcm12093323/s1, Figure S1: Funnel plot for GERD and BMI in patients assigned to SG + HHR; Figure S2: Funnel plot for GERD and BMI in patients assigned to SG + FP.
