# Supplementary material for: Efficacy of Sleeve Gastrectomy with Concomitant Hiatal Hernia Repair versus Sleeve–Fundoplication on Gastroesophageal Reflux Disease Resolution: Systematic Review and Meta-Analysis
Source: J Clin Med. 2023 May 6;12(9):3323. doi: 10.3390/jcm12093323 (PMC10179224; doi:10.3390/jcm12093323)
Supplement: Supplementary file 1 [file jcm-12-03323-s001.zip › jcm-2326632-supplementary.pdf]

Supplementary material

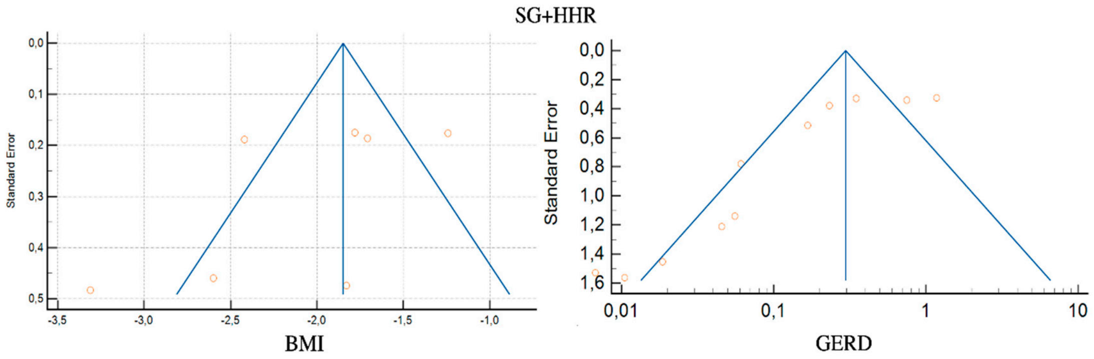

Supplementary figure S1. Funnel plot for GERD and BMI in patients assigned to SG+HHR

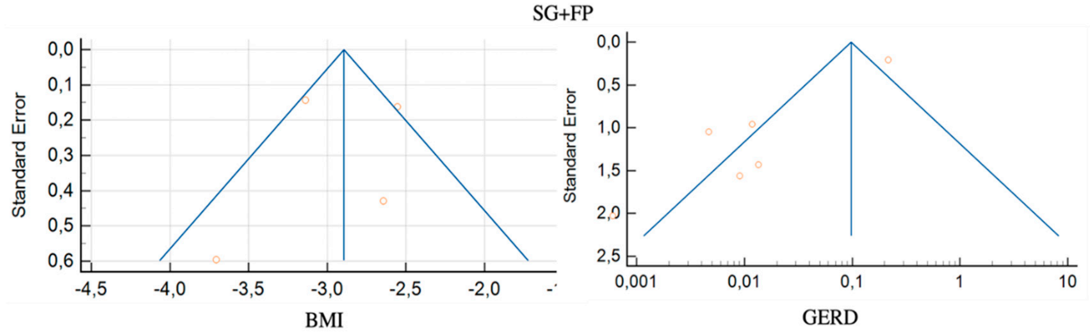

Supplementary figure S2. Funnel plot for GERD and BMI in patients assigned to SG+FP
